# From an Open Bite to a Harmonious Smile: Orthodontic Intervention With Bluegrass Appliance and Tongue Thrust Resolution

**DOI:** 10.7759/cureus.61024

**Published:** 2024-05-24

**Authors:** Mrudula Shinde, Pallavi Daigavane, Ranjit Kamble, Nishu Agarwal, Dhwani Suchak, Aathira Surendran, Utkarsha Chaudhari, Aditya V Pareek

**Affiliations:** 1 Orthodontics and Dentofacial Orthopedics, Sharad Pawar Dental College and Hospital, Datta Meghe Institute of Higher Education and Research, Wardha, IND

**Keywords:** positive reinforcement, bluegrass appliance, tongue posture, tongue thrusting, anterior open bite

## Abstract

The tongue-thrusting habit significantly contributes to the development of the anterior open bite (AOB), particularly when an infantile swallowing pattern persists into the later stages of childhood and adolescence. This habit results in the protrusion of the anterior teeth. Treatment typically involves addressing the underlying causes, incorporating retraining exercises, and utilizing mechanical appliances to control tongue positioning. However, commonly used devices such as palatal cribs or spurs may present challenges, including speech impediments, chewing difficulties, and the potential for unintended injuries. This paper presents a case report detailing the treatment of a patient with an AOB, dental protrusion, and spacing. The treatment approach included the application of a fixed tongue trainer, in the form of a modified bluegrass appliance. Subsequent fixed orthodontic therapy was employed to rectify proclined teeth within the dental arch.

## Introduction

Orthodontics is the study of dental and oral development, aiming to identify the factors that control growth processes to achieve a normal, functional, and anatomical relationship of these parts and to understand the influences necessary to maintain these conditions once established. Malocclusion can occur in three planes of space that is sagittal, transverse, and vertical plane. In 1842, Caravelli coined the term "open bite" to distinguish between various types of malocclusion [1]. He introduced a distinct classification that differed from those previously defined by other scholars. According to Lefonlon's research (1841), external muscular stresses exerted by the lips and cheeks were identified as the primary cause of abnormalities, while internal muscle forces, particularly the tongue and occlusal forces, were also considered [2]. An "anterior open bite" (AOB) is the absence of incisal contact between the anterior teeth in centric relation [3].

An AOB of more than 2 mm is a condition seen in less than 1% of the general population. However, this condition is five times more prevalent in the Black population than in the White or Hispanic populations. The prevalence of AOB varies across racial groups and dental age, ranging from 1.5% to 11%. During the mixed dentition era, the prevalence of AOB can reach up to 18.5% and then gradually decrease with age. These statistics may be of interest to those involved in the field of dentistry [4].

It has been noted that the positioning of the tongue at rest plays a significant role than its posture during swallowing in determining the shape of the dental arch. An anterior resting position of the tongue, applying mild and continuous pressure against the teeth, is expected to impact both the vertical and horizontal alignment of the teeth. This impact is thought to play a role in initiating and sustaining AOBs. In the typical resting state, the tongue's tip rests on the incisive papilla, and its back lies along the palate, ensuring the maintenance of anterior occlusion and the transverse dimension of the upper arch [5].

Various hypotheses have been put forth to explain the development of an open bite, encompassing factors such as heredity, unfavorable growth patterns, digit suckling habits, enlarged lymphatic tissue, the function and posture of the tongue, orofacial muscle activity, imbalance between jaw posture, occlusal and eruptive forces, and head position [2].

Certain researchers propose that the size and dysfunction of the tongue are fundamental factors contributing to the development of malocclusion, while others argue that tongue thrust swallowing should be considered an effect rather than a cause of malocclusion. Their perspective is grounded in the notion that in the presence of an overjet or open bite, achieving a proper seal at the front of the mouth during swallowing becomes challenging. The treatment strategy for these habits includes eliminating the underlying cause, incorporating retraining exercises, and utilizing mechanical restraining appliances [5].

## Case presentation

A 19-year-old female patient presented to the Department of Orthodontics and Dentofacial Orthopedics at Sharad Pawar Dental College and Hospital, citing a chief complaint of upper front teeth protrusion. A comprehensive habit history and clinical assessment were conducted, accompanied by the collection of the patient's pretreatment records, which included radiographs, study models, and photographs.

**Figure 1:**
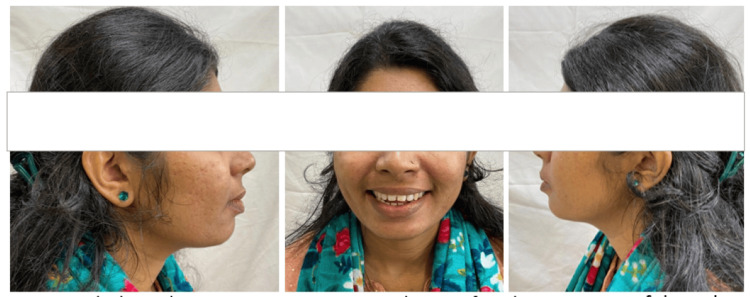
Pretreatment extraoral photograph (A) Right lateral at rest, (B) frontal view of the smile, (C) left lateral at rest

The extraoral assessment of the patient revealed a convex profile, an average nasolabial angle, a well-balanced facial symmetry, a shallow mentolabial sulcus, and lips at rest displaying incompetence (Figure 1).

**Figure 2:**
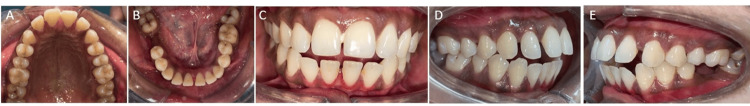
Pretreatment intraoral photographs (A) Maxillary occlusal, (B) mandibular occlusal, (C) frontal view of occlusion, (D) right lateral view of occlusion, (E) left lateral view of occlusion

Intraoral examination indicated class I molar relationship on the right side of the arch and class I canine relationships on both sides, inclination toward an open bite, forwardly positioned upper and lower anterior teeth, an increased overjet in the range of 4-6 mm along with primarily oral mode of respiration (Figure 2). On the functional examination, the patient showed compensatory tongue thrust with poor pronunciation of sibilant and fricative sounds. A thorough investigation into the patient's tongue habit history revealed that the patient habitually placed her tongue forward against the front teeth during swallowing. She was diagnosed with a simple tongue thrust habit, which caused generalized spacing in both the upper and lower dental segments. This resulted in a class I dental open bite malocclusion, characterized by a symmetrical 3 mm open bite restricted to the incisor and right canine region.

**Figure 3:**
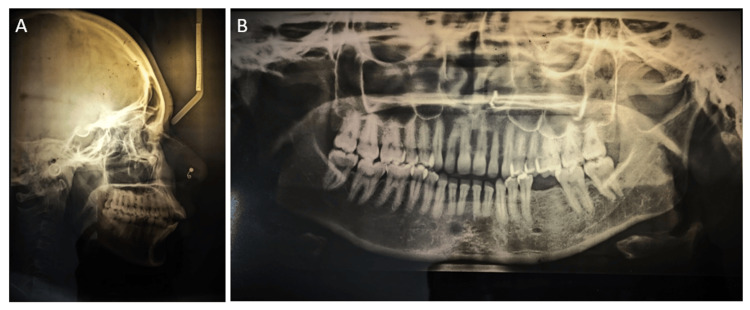
Pretreatment radiographs. (A) Lateral cephalogram, (B) orthopantomogram

The radiographic analysis revealed skeletal class II bases with horizontal growth pattern, forwardly positioned maxillary and mandibular central incisors, an acute nasolabial angle, and cervical vertebrae maturation index stage VI (maturation) (Figure 3).

A diagnosis of tongue thrusting was established following a clinical examination and review of the patient's history. The patient exhibited a complete set of permanent teeth, except the mandibular left first molar, which was absent due to severe carious destruction and had been extracted by the patient's dentist 5-6 years back.

The principal objective of the treatment was to eliminate the underlying cause, which, in this case, was the forward tongue resting posture. The goal was to attain a satisfactory occlusion, close any existing spaces, establish an ideal overjet and overbite, and align the midlines. This involved appropriately aligning the teeth and rectifying the forward positioning of the maxillary and mandibular incisors. The aim was to achieve optimal facial aesthetics, balance, and a harmonious smile.

The proposed treatment approach uses fixed mechanotherapy to retract both upper and lower incisors while incorporating a composite habit-breaking appliance to alleviate tongue habit in the first phase. To address the critical anchorage issue in both the upper and lower arches, we initiated treatment using a preadjusted edgewise appliance (MBT 0.022” bracket slot, provided by Libral Traders, New Delhi, India). Alignment began with a 0.016” nickel-titanium wire in both the maxillary and mandibular arches, progressing to a 0.017 × 0.025’’ SS wire over a four-month alignment period.

**Figure 4:**
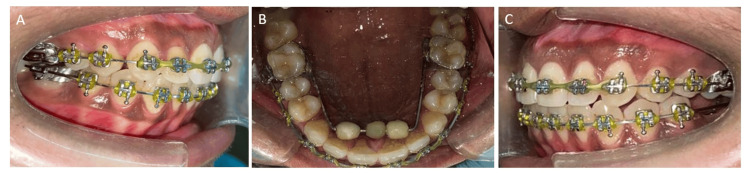
Intraoral stage record (A) Right lateral view of occlusion, (B) maxillary occlusal, (C) left lateral view of occlusion

To correct the AOB and mitigate tongue-thrusting habits, a bluegrass appliance was employed. The patient received training to move the bead towards the back, aiming to retrain the tongue to adopt a posture away from the front teeth (Figure 4) [6].

**Figure 5:**
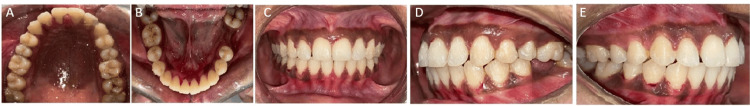
Posttreatment intraoral photograph (A) Maxillary occlusal, (B) mandibular occlusal, (C) frontal view of occlusion, (D) left lateral view of occlusion, (E) right lateral view of occlusion

Following the completion of the case, the patient's primary concern regarding irregularly placed and proclined anterior teeth was addressed. The outcome includes a harmonious smile, satisfactory lip competency, retraction of the upper anterior, and the maintenance of class I molar relations on the right side (Figure 5).

**Figure 6:**
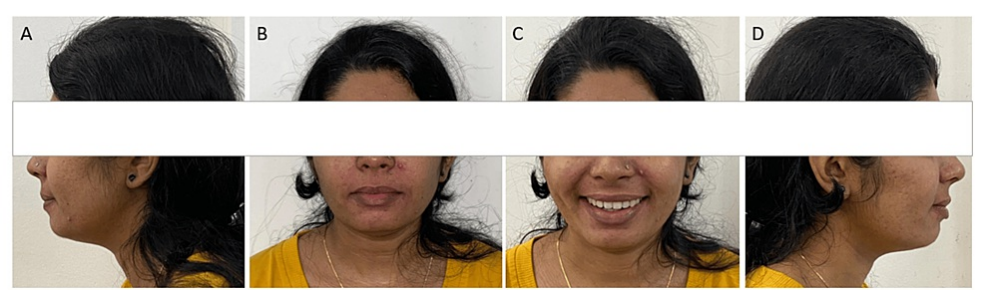
Posttreatment extraoral photograph (A) Left lateral at rest, (B) frontal view at rest, (C) frontal view of smile, (D) right lateral at rest

Ideal overjet and overbite, along with optimal facial aesthetics and balance, were achieved. The pre- and posttreatment photographs and radiographs revealed a significant correction of tongue thrusting and notable improvements in profile, overjet, and overbite (Figure 6). Additionally, the treatment resulted in enhanced smile aesthetics and rectification of proclination in the upper and lower anterior teeth.

## Discussion

Tongue thrusting, a condition where the tongue protrudes through the anterior teeth during swallowing due to an improper resting position, is classified into two types: primary and secondary. Primary tongue thrusting can occur due to acquired behavior, prolonged thumb sucking, or nasal obstruction. On the other hand, secondary tongue thrusting often arises due to deciduous teeth extraction or an AOB [7]. The conventional clinical treatment of tongue thrusting typically involves addressing each issue separately using equipment sequentially. In this case report, the bluegrass appliance was used to address both tongue thrusting and AOB. It is worth noting that spike, crib, and rake appliances, although used for a similar purpose, use a punitive method rather than a positive reinforcement approach [8].

Therefore, in this case report, a positive reinforcement approach was employed, utilizing a bluegrass appliance equipped with a roller. Positioned at the highest point of the palate, the roller does not interfere with eating, emotional well-being, or inadvertently causing self-inflicted wounds. Additionally, it minimally disrupts speech. The design of the appliance's roller adheres to principles of positive reinforcement, operating via a counterconditioning response to the initial conditioned stimulus associated with tongue pushing [9].

In this case, a roller device was utilized as a corrective measure for the issue of tongue pushing behavior and AOB. According to Zameer et al., the roller can be considered a viable therapeutic technique to prevent sucking habits and address dentofacial deformities. In this particular instance, it was employed to correct the problem of tongue-thrusting tendency and AOB [10].

## Conclusions

The present case report reiterated the significance of anterior tongue posture in the overall management strategy of open bite malocclusion. Determining the type of resting posture of the tongue and its targeted addressed while planning mechanotherapy is of paramount importance to ensure long-term stability. To mitigate the demand for patient compliance toward tongue exercise instructions, it is recommended to stimulate the hind section of the tongue.
